# A Multiplex PCR Melting-Curve-Analysis-Based Detection Method for the Discrimination of Five *Aspergillus* Species

**DOI:** 10.3390/jof9080842

**Published:** 2023-08-11

**Authors:** Maria Tokamani, Eleftheria Figgou, Lito Papamichail, Eleni Sakka, Athanasios Toros, Anastasia Bouchorikou, Antonis Giannakakis, Efthymia Iliana Matthaiou, Raphael Sandaltzopoulos

**Affiliations:** 1 Department of Molecular Biology and Genetics, Faculty of Health Sciences, Democritus University of Thrace, 68100 Alexandroupolis, Greece; tokamanimaria@hotmail.com (M.T.); elefigg@maich.gr (E.F.); l.papamichail@erasmusmc.nl (L.P.); eleni.a.sakka@gmail.com (E.S.); athanasiostoros@gmail.com (A.T.); anastasia.bouhorikou@gmail.com (A.B.); antgian@mbg.duth.gr (A.G.); 2 Department of Medicine, Division of Pulmonary Allergy and Critical Care Medicine, School of Medicine, Stanford University, Stanford, CA 94305, USA; matthaiou.evily@gmail.com

**Keywords:** *Aspergillus fumigatus*, *Aspergillus flavus*, *Aspergillus niger*, *Aspergillus terreus*, *Aspergillus nidulans*, detection, SYBR Green I, mRT-PCR, melting curve analysis, ITS

## Abstract

*Aspergillus* mold is a ubiquitously found, airborne pathogen that can cause a variety of diseases from mild to life-threatening in severity. Limitations in diagnostic methods combined with anti-fungal resistance render *Aspergillus* a global emerging pathogen. In industry, *Aspergilli* produce toxins, such as aflatoxins, which can cause food spoilage and pose public health risk issues. Here, we report a multiplex qPCR method for the detection and identification of the five most common pathogenic *Aspergillus* species, *Aspergillus fumigatus*, *Aspergillus flavus*, *Aspergillus niger*, *Aspergillus terreus*, and *Aspergillus nidulans.* Our approach exploits species-specific nucleotide polymorphisms within their ITS genomic regions. This novel assay combines multiplex single-color real time qPCR and melting curve analysis and provides a straight-forward, rapid, and cost-effective detection method that can identify five *Aspergillus* species simultaneously in a single reaction using only six unlabeled primers. Due to their unique fragment lengths, the resulting amplicons are directly linked to certain *Aspergillus* species like fingerprints, following either electrophoresis or melting curve analysis. Our method is characterized by high analytical sensitivity and specificity, so it may serve as a useful and inexpensive tool for *Aspergillus* diagnostic applications both in health care and the food industry.

## 1. Introduction

*Aspergillus* species are omnipresent and exhibit a great degree of diversity. Due to their high adaptability, they can grow on various surfaces and thrive in a variety of widely different climates and environments that differ vastly in temperature, oxygen availability, humidity, and pH. *Aspergillus* hyphae produce conidiophores that release thousands of airborne spores (conidia), which can easily spread at long distances [1].

Daily exposure to *Aspergillus* species is unavoidable but rarely a problem for people with healthy immune systems [2]. Depending on the underlying state of the human immune system, *Aspergillus* conidia can cause a variety of diseases that range in severity. The clinical spectrum of diseases associated with *Aspergillus* species include atopic asthma, hypersensitivity pneumonitis, allergic bronchopulmonary aspergillosis (ABPA), aspergilloma, and the life-threating invasive aspergillosis (IA) [3]. According to the United States Centers for Disease Control and Prevention (CDC), ABPA affects 15% of cystic fibrosis patients, and 2.5% of adults with asthma also develop ABPA, which corresponds to approximately 4.8 million people worldwide [4]. In immunocompromised patients (e.g., solid organ or stem cell transplant patients), *Aspergillus* can cause severe or life-threatening infections of the sinuses or lungs, which can spread to other parts of the body [5]. Furthermore, there is increasing evidence that invasive *Aspergillus* infections are associated with viral and bacterial pulmonary infections (including but not limited to influenza, coronavirus, pseudomonas, and mycobacterium tuberculosis) and lung cancer [6,7,8,9,10,11,12,13,14,15].

Despite its high occurrence, aspergillosis is one of the leading missed diagnoses in intensive care units. This is due to the limitations of the current diagnostic methods. Moreover, until recently clinicians had limited access to anti-fungal susceptibility testing [16]. Since the severity of the *Aspergillus*-related diseases depends on the *Aspergillus* strain, it is pivotal to identify the stain and the right anti-fungal treatment for each case [17].

Out of the over 250 known species of *Aspergillus*, fewer than 40 are known to cause human infections. *Aspergillus fumigatus* (*A. fumigatus*) is the most common etiologic agent for IA and is responsible for about 90% of IA cases. Other pathogens in the genus that could cause IA are *A. flavus*, *A. niger*, *A. terreus*, *A. lentulus*, *A. ustus*, *A. glaucus*, and *A. nidulans* [1,18]. *Aspergillus* species are frequently found in hospitals, mainly in ventilation and water systems. The vast majority of *Aspergillus* strains identified in such cases belong to the species *A. fumigatus*, *A. flavus*, *A. niger*, *A. terreus*, and *A. nidulans*. There is evidence that the presence of these species in hospital premises increases the risk of aspergillosis in hospitalized patients [19,20,21]. Early diagnosis of IA and identification of the *Aspergillus* species could be lifesaving [22,23].

Aspergilli also infect plant seeds and groundnuts, leading to crop failure and decay [24]. In addition, due to prevailing agricultural practices and food storage, grains, cereals, and cereal-based products retain substantial moisture content (>14%), which creates an ideal environment for fungal growth [25]. *A. flavus*, *A. niger*, *A. oryzae*, *A. ochraceus*, *A. parasiticus*, and other species are responsible for the production of highly toxic secondary metabolites called mycotoxins, such as aflatoxins and ochratoxins [26]. Industrial food processing usually fails to eliminate mycotoxins adequately because these toxins are heat resistant [25,27]. Since many types of fruits, vegetables, meat, and spices are ideal environments for fungal growth, measures must be taken to avoid mycotoxicosis caused by the consumption of contaminated food products [26]. It is essential to monitor food products for *Aspergillus*’ contamination pre- and post-harvest, as well as along storage and industrial processing steps. Having a sensitive, straight-forward, rapid, and cost-effective method to detect *Aspergillus* species may serve as a good practice towards minimizing public health risk and produce/food spoilage [25]. There is a clear need for the development of novel detection methods that will allow us to distinguish the *Aspergillus* species. High-throughput, yet simple, reliable, and cost-effective, molecular testing methods are crucial for the detection of microorganisms [28]. Among the many valuable tools that have been explored in nucleic-acid-based testing, real-time polymerase chain reaction (qPCR)-based methods have unique advantages because they allow real-time, kinetic detection of amplified product accumulation through fluorescence intensity change in a closed-tube setting, eliminating the need for post-amplification manipulation, and thereby markedly reducing the chances of cross-contamination [29]. qPCR techniques represent an excellent alternative to existing standard culture methods as they enable reliable detection and quantification for several pathogens [30]. Multiplex qPCR methods present advantages over the conventional qPCR since they allow (i) simultaneous detection of a few microorganisms in a single reaction and (ii) reduction of the use of PCR samples [31]. For the detection of multiple pathogens, probe-based qPCR is preferred. There are various fluorescent probes developed for this kind of analysis, including hydrolysis (TaqMan) probes, molecular beacons, dual hybridization probes, eclipse probes, amplifluor assays, and scorpion probes, as well as LUX probes and QZyme probes [32,33,34,35,36,37]. These probes are highly sensitive, sequence-specific, and fluorescently labeled, but their key disadvantage is the oligo design complexity.

Here, we present an innovative *Aspergillus* detection method, which employs multiplex qPCR as well as melting curve analysis to detect and identify the most common pathogenic *Aspergillus* species (*A. fumigatus*, *A. flavus*, *A. niger*, *A. terreus*, and *A. nidulans*). Our method directly identifies five *Aspergillus* species in a single reaction, which has obvious advantages compared to the previously described molecular detection methods [38,39]. To our knowledge, this is the first time that a multiplex qPCR melting curve analysis method has been designed for the simultaneous detection and discrimination of these five *Aspergillus* species.

## 2. Materials and Methods

### 2.1. Fungal Species and Biological Material

Genomic DNA from five *Aspergillus* species was obtained from the CBS-KNAW culture collection of the Westerdijk Fungal Biodiversity Institute. The selected strains were *Aspergillus nidulans* (CBS 589.65), *Aspergillus fumigatus* (CBS 113.26), *Aspergillus niger* (CBS 118725), *Aspergillus flavus* (CBS 542.69), and *Aspergillus terreus* (CBS 134.60). Additionally, DNA extraction was performed from human sputum samples spiked with *Aspergillus* DNA using Macherey-Nagel™ NucleoSpin™ Soil (Düren, Germany, Cat.no. 740780).

Previously identified and characterized direct isolates were used to test the efficiency of our method. Specifically, two *A. fumigatus*, two *A. flavus*, three *A. niger*, and two *A. terreus* colonies were isolated from sunflower field soil samples (collected in the region of Evros, Greece) after dilution in sterile water and culture at 28 ± 2 °C for 5–7 days in Glucose-Czapek’s agar medium [40]. The morphological and micromorphological characterization of isolated colonies was performed following the procedures of Klich and Pitt (1988) [41] and Samson et al. (2014) [42].

### 2.2. Primer Design

**Figure 1:**
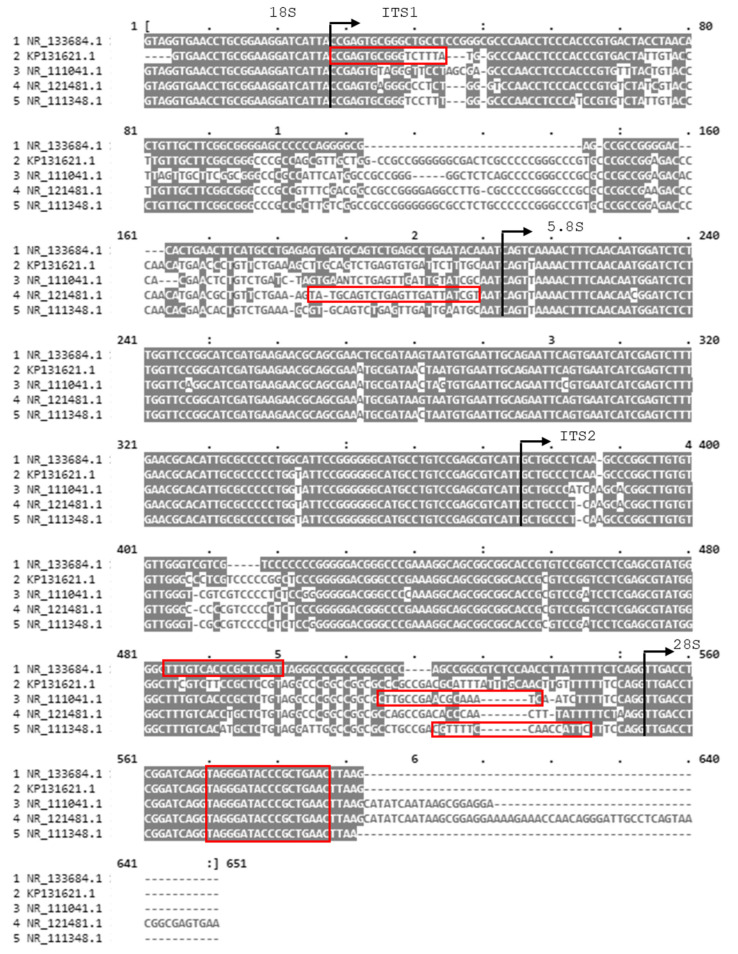
MUSCLE multiple sequence alignment of the ITS regions of the five *Aspergillus* species: *A. nidulans* (NR_133684); *A. terreus* (KP131621); *A. flavus* (NR_111041); *A. fumigatus* (NR_121481); and *A. niger* (NR_111348). The red rectangles highlight the location of the five forward primers and the common reverse primer. The vertical lines with the right-facing arrowhead denote the start of the ITS1, 5.8S, ITS2, and 28S regions respectively.

To detect and differentiate the selected species, ITS (internal transcribed spacer) regions of the ribosomal RNA gene cluster were used as markers due to the presence of inter-species variations. We selected species-specific sequence variations within the ITS regions that may serve as diagnostic landmarks allowing discrimination of *Aspergillus* species [43]. In order to develop a multiplex qPCR assay using a minimum set of primers, one consensus reverse primer and five species-specific forward primers were designed. rRNA genes from the GenBank database were aligned using the MUSCLE algorithm (Figure 1). A conserved motif inside the LSU region (large subunit ribosomal RNA region) was used to design a consensus common reverse primer, while the polymorphic regions ITS1 and ITS2 were used to design species-specific forward primers. The specificity of the primers and their potential cross-reaction with other fungi was examined in silico using the Basic Local Alignment Search Tool (Primer-BLAST and BLASTn) by NCBI. Additionally, the formation of primer dimers was tested in silico using the PerlPrimer tool. The melting temperatures (Tm) of the expected amplicons were adjusted to differ by at least one degree, using the uMELT tool. The final sequences of the selected primers were synthesized by Eurofins Genomics (Ebersberg, Germany) and are demonstrated in Table 1.

### 2.3. qPCR Reaction

Real-time PCR (qPCR) was performed on a StepOne PCR System in MicroAmp^®^ Fast Optical 48-well reaction plates (Applied Biosystems, Waltham, MA, USA) using KAPA SYBR^®^ FAST Universal Kit [KK4601] (Sigma-Aldrich, St. Louis, MO, USA). The reaction was optimized, and each reaction mixture included: (i) 10 μL of 2× KAPA SYBR Fast qPCR Universal buffer, (ii) 0.4 μL High ROX dye, (iii) 1 μL diluted genomic DNA (typically 10 ng of DNA), (iv) 0.2 μM of the reverse primer, (v) 0.3 μM of *A. niger*-specific forward primer, and (vi) 0.18 μM of each of the rest of the species-specific forward primers. The amplification process was conducted in a bi-phase protocol as follows: initial denaturation at 95 °C for 2 min, a first phase of 5 cycles of denaturation at 95 °C for 5 s, and primer annealing and extension at 70 °C for 30 s, which was followed by a second phase of 30 cycles of denaturation at 95 °C for 5 s, and primer annealing and extension at 60 °C for 30 s.

Each experiment included two no-template controls for each primer mix. The reproducibility of the method was checked by performing each reaction at least six times. PCR products were evaluated by melting curve analysis using the following steps: denaturation at 95 °C for 15 s, annealing at 60 °C for 1 min, and gradual denaturation from 60 °C to 95 °C by measuring fluorescence emission per 0.3 °C every 15 s. At the end, PCR products were resolved and visualized by electrophoresis on 2% *w*/*v* agarose gel containing ethidium bromide (EtBr, 0.5 μg/mL) in 0.5× TBE buffer and observed under UV light (ChemiDoc Imaging Systems, Life Science Research, Bio-Rad, Hercules, CA, USA).

### 2.4. Efficiency and Sensitivity of the Method

The sensitivity of the multiplex qPCR method was checked by using seven 10-fold serial dilutions of each DNA template (100 ng/μL to 0.1 pg/μL). Each one of the serial diluted DNA templates was assayed in duplicates along with two no-template controls. The multiplex qPCR linear dynamic ranges of each *Aspergillus* DNA sample were 10 ng to 1 pg for *A. terreus* and *A. nidulans*, and 10 ng to 10 pg for *A. fumigatus*, *A. flavus*, and *A. niger*. The Ct of the dilutions on the dynamic ranges of each template was used to calculate the slope of the linear regression between Ct values (y-axis) and DNA concentrations (x-axis), the efficiency $E\left(\%\right)=\left(\left(10^{\left[-\frac{1}{slope}\right]}\right)-1\right)\times 100$, and the squared regression coefficient (R^2^) for each template.

### 2.5. Specificity of the Method

The specificity of each forward primer was tested by qPCR reactions. The positive control qPCR reactions included the consensus reverse primer (0.2 μM), one of the species-specific forward primers (0.2 μM), and the targeted template (10 ng). Two no-template reactions for each primer set served as negative controls. To test for cross-reactivity of the different sets of primers, a third type of reaction was designed. This qPCR included the consensus reverse primer (0.2 μM) and one of the species-specific forward primers (0.2 μM) with 10 ng of each of all DNA templates except for the corresponding one (i.e., a total 40 ng of template mix). Each reaction was performed in duplicates.

### 2.6. In Silico Evaluation of Primers

Consensus sequence creation: The proposed primer sets were evaluated in silico based on their ability to selectively amplify the specified *Aspergillus* species. To achieve this, a total of 5776 sequences containing the ITS2 and LSU regions were downloaded from the NCBI Genbank database, using the following search query: Aspergillus (organism) AND (“ITS2” (all fields) OR “ITS1” (all fields) OR “ITS” (all fields)) AND (“LSU” (all fields) or “28s” (all fields). All sequences were aligned using the MUSCLE [44] plugin from UGENE [45] with the default settings and were subsequently trimmed using the KP131621.1 (*A. terreus*) sequence as a guide. Following this, the MetaCurator toolkit [46] was employed to extract and dereplicate the region of interest for each sequence. The five sequences utilized in the creation of the primer set were set as the reference for the default pipeline of this tool, with the iterationseries and coverageseries arguments being set to “6,6,3,3,3” and “1.0,0.98,0.95,0.9,0.85”, respectively. In total, 1408 sequences remained after this step.

In order to create a reference sequence for each species, the sequences of the previous step were matched first to a taxid using the list provided by NCBI at https://ftp.ncbi.nlm.nih.gov/pub/taxonomy/accession2taxid/nucl_gb.accession2taxid.gz (accessed on 7 July 2023), and then to the appropriate species using TaxonKit [47]. Afterwards, the sequences for each species were split, and then aligned using MUSCLE. Finally, the consensus sequences for each species were calculated using the corresponding UGENE command line workflow, with the “Simple extended” algorithm and a threshold of 35%. Leading and trailing nonspecific bases were also trimmed from the final sequences.

In silico PCR was performed for each primer set against the consensus sequences of all species, using thermonucleotideBLAST [48]. The resulting hits were limited to a maximum of three mismatches with the template, with none of them being at the 3′ end of the primer.

Phylogenetic Tree: Aiming to better understand the selectivity of the proposed primers for closely related species, a phylogenetic tree was constructed for the consensus sequences used in the in silico PCR. The alignment of the sequences was performed using MAFFT v7.490 [49,50], with a gap opening penalty of 1.53. Additionally, the masking algorithm from the QIIME2 [51] toolkit was used to improve the phylogenetic accuracy of the alignment (min conservation = 0.4, max gap frequency = 0.95). In the final step, the phylogenetic tree was calculated using the fasttree plugin from QIIME2 [52] and visualized using the iTOL toolset [53].

## 3. Results

### 3.1. Optimization of Multiplex Real-Time PCR

In order to detect and identify the five *Aspergillus* species (*A. terreus*, *A. flavus*, *A. fumigatus*, *A. nidulans*, and *A. niger*), rRNA gene were aligned to detect polymorphic regions among them. As presented in Figure 1, a set of one consensus reverse primer and five species-specific forward primers were designed, to amplify PCR products with distinct molecular weight and Tm.

**Figure 2:**
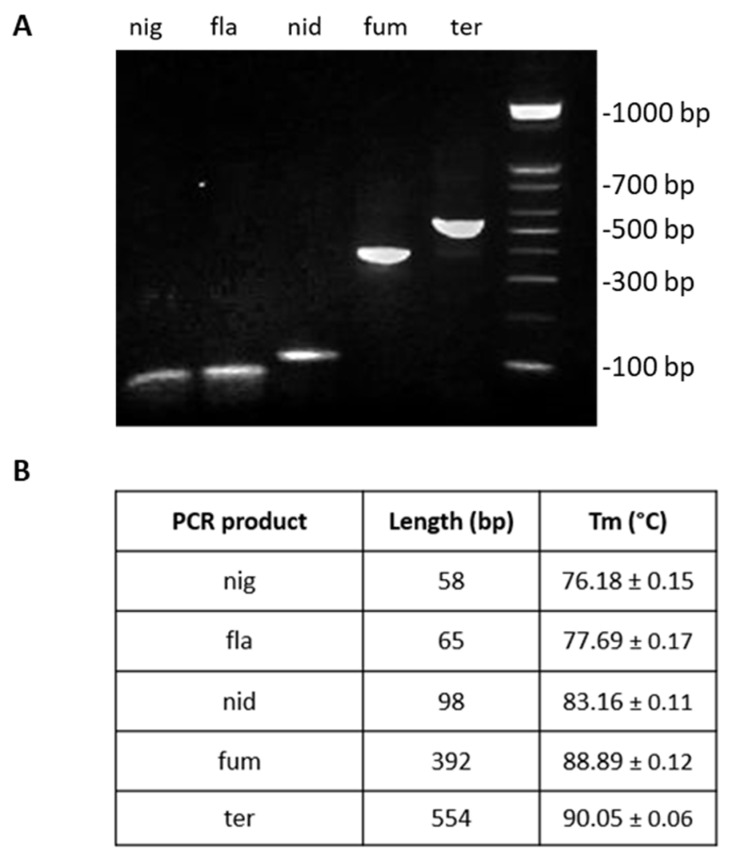
Gel electrophoresis of qPCR reaction products using a specific diagnostic primer set with the corresponding template (**A**) and the Tm values and fragment sizes (**B**) for the species *A. niger* (*nig*), *A. flavus* (*fla*), *A. nidulans* (*nid*), *A. fumigatus* (*fum*), and *A. terreus* (*ter*).

DNA samples from the five *Aspergillus* species provided by the CBS-KNAW culture collection of the Westerdijk Fungal Biodiversity Institute were tested by real-time PCR with a single primer set each in order to confirm that all primer sets amplified a unique product with distinct molecular weight specifically (Figure 2A) and that no product was generated when non-specific templates were used.

**Figure 3:**
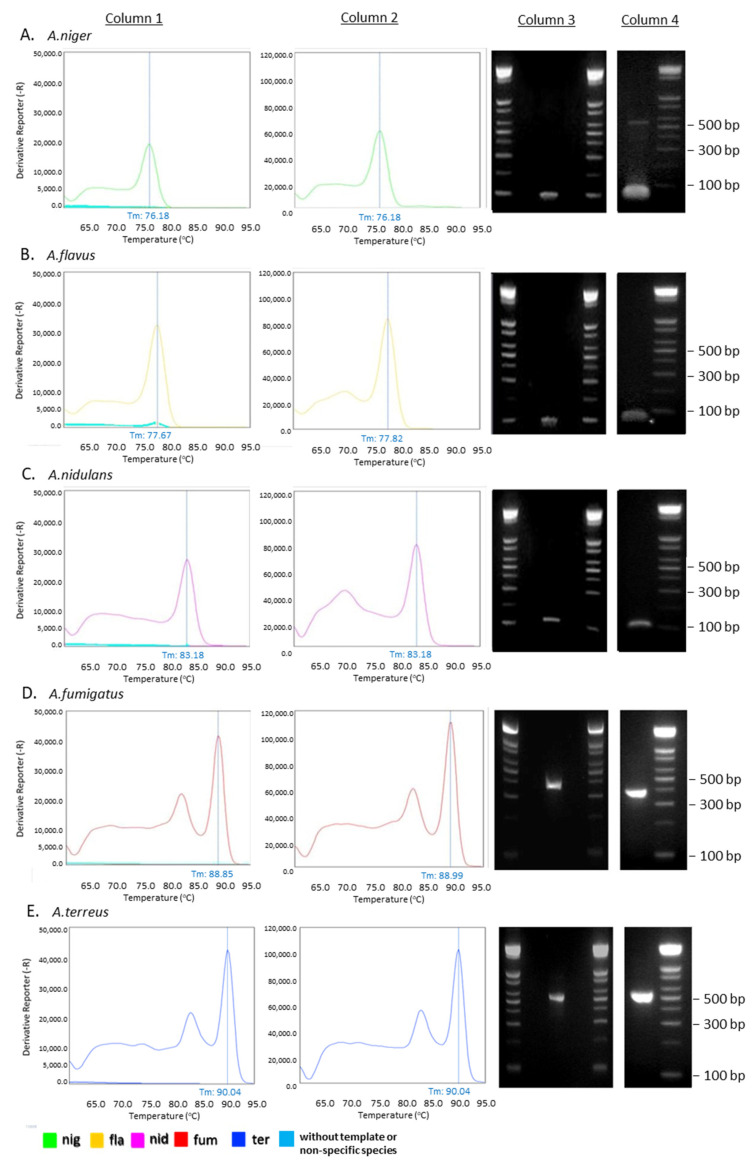
Melting curve analysis and gel electrophoresis of qPCR products. Column 1: Single-species-specific qPCR reactions. Each reaction utilized a unique set of haplotype-specific primers designed exclusively for the detection of one *Aspergillus* species. Three reactions were performed for each primer pair: the first one without any template, the second one with the corresponding DNA template, and the third one with all the other four non-specific templates ((**A**) *A. niger*, (**Β**) *A. flavus*, (**C**) *A. nidulans*, (**D**) *A. fumigatus*, and (**E**) *A. terreus*). Column 2: Multiplex real-time PCR reactions combining all five primer using as template DNA isolated from a single *Aspergillus* species. Column 3: Gel electrophoresis analysis of single-species-specific qPCR reactions (lane 1: ladder, lane 2: no template control, lane 3: corresponding species DNA template, lane 4: non-specific templates, lane 5: ladder). Column 4: Gel electrophoresis analysis of multiplex real-time PCR reaction using all five sets of primers.

The length of the expected PCR amplicons and the corresponding Tm values as determined by the melting curve analysis are shown on Figure 2B. Tm values of the species-specific products differ by at least 1 °C, which allowed us to easily identify each one of the five *Aspergillus* species by melting curve analysis. Gel electrophoresis of the species-specific PCR amplicons is presented in Figure 2 and Figure 3A–E (column 3).

Optimized multiplex qPCR reactions (one template per reaction) lead to single-band species-specific products of expected lengths (Figure 3A–E, column 4). In all cases, the multiplex reactions for each species yielded an identical melting curve to the reaction in which a single set of primers was used, indicating that the primer mix is appropriate for the amplification of any of the five *Aspergillus* species in a multiplex reaction. Since the PCR products corresponding to different species have different lengths, gel electrophoresis analysis can be performed to confirm the identification of each species.

It is worth noting that detection and species discrimination by our multiplex PCR was successful even when a template of suboptimal quality was used (e.g., DNA extracted from spiked-in sputum samples).

Last but not least, we simulated co-infection situations by combining pairs of DNA templates (Supplementary Table S1) in the multiplex real-time PCR. Under such conditions of primer competition, we found that species with higher primer Tm values were able to mask detection of other species unless DNA of a less potent species was present at higher relative concentration (i.e., detection potency followed the order: *A. terreus* > *A. fumigatus* > other three species).

### 3.2. Sensitivity of Multiplex qPCR

**Figure 4:**
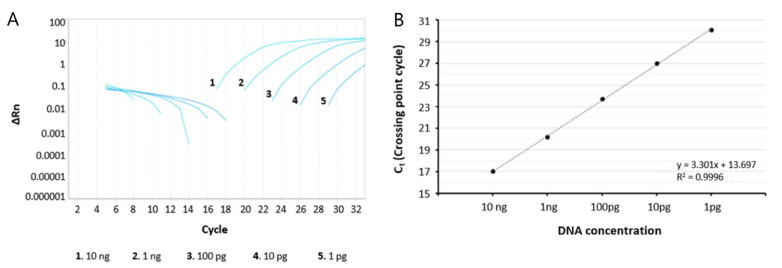
Sensitivity assay: ΔRn—cycle diagram (**A**) and standard curve diagram (**B**) of exemplary real-time PCR reactions using serial template dilutions (10 ng to 1 pg) amplified by the specific set of primers for *A. terreus*. Similar results were obtained with all *Aspergillus* species and the corresponding specific diagnostic primer set.

According to the MIQE guideline, analytical sensitivity is the minimum number of copies that can be measured accurately with an assay [54]. Based on the median total genome lengths (NCBI-Genome database, https://www.ncbi.nlm.nih.gov/genome, accessed on 15 March 2023; *A. fumigatus* 28.83 Mb; *A. niger* 35.63 Mb; *A. nidulans* 30.05 Mb; *A. flavus* 36.81 Mb; *A. terreus* 29.36 Mb), we determined the approximate number of genome copies required per reaction in order to yield a detectable signal. Approximately 10 ng of each DNA template corresponded to 3 × 10^5^ genome copies. We used seven 10-fold serial dilutions of each DNA template (100 ng down to 0.1 pg, which is equal to 3 × 10^6^ down to 3 genome copies, based on the calculator for determining the number of copies of a template from the URI Genomics & Sequencing Center) to evaluate the analytical sensitivity of single and multiplex qPCR. Amplification curves and standard curves of the dynamic range of concentrations of multiplex qPCR showed that even 1 pg (~30 genome copies) was sufficient to yield a readily detectable signal in the case of *A. terreus*, *A. fumigatus*, and *A. nidulans*, while in the case of *A. flavus* and *A. niger*, 10 pg (~300 copies) was required (Figure 4, using as template DNA derived from *A. terreus*; very similar results were obtained with all *Aspergillus* DNA templates).

In order to further test our primer sets’ sensitivity in the context of intra-species genetic variation, at least two isolates were tested per species, except for *A. nidulans*, due to a lack of available isolates. As expected, each isolate was correctly assigned to the corresponding species with our methodology. The assessment of the *A. nidulans*-specific primer was based on a thorough in silico analysis of publicly available sequences. After discarding a minimal number of low-quality sequences, our primer set demonstrated full sensitivity (Supplementary Material S1).

### 3.3. Specificity

The set of multiplex primers was submitted to rigorous specificity tests to eliminate the possibility of cross-reactivity, which could lead to false positive results. Each primer set was used in a reaction containing DNA templates derived from all *Aspergillus* species except that one corresponding to the particular primer set. No PCR product was produced under these conditions (negative control) as all sets of primers yielded a fragment of the expected Tm value only when the specific template was present (see melting curve analysis in Figure 3A–E, columns 1 and 2). The fragment identity was confirmed by analysis of its expected length in an agarose gel electrophoresis (Figure 3A–E, columns 3–4). In the case of *A. niger*, *A. flavus*, and *A. nidulans*, a single peak was detected by melting curve analysis. In the case of the species *A. fumigatus* and *A. terreus*, melting curve analysis revealed a double peak. This pattern involving a second lower peak accompanying that one corresponding to the full-length fragment was reproducible, and the ratio of the heights of the two peaks was always constant, indicating that the minor peak could be attributed to the presence of certain secondary structures in the amplicon regions of the full-length PCR products (>150 bp). Moreover, neither the reactions containing no template nor the reactions with all the templates except for the specific one yielded any peak, indicating that no unspecific products were generated. In conclusion, all five sets of primers yield a single product exclusively in the presence of the specific template. Gel electrophoresis of the reaction products confirmed this conclusion as the fragment lengths detected corresponded with the melting curve analysis peaks in all cases.

We performed in silico analysis in order to evaluate primers’ ability to selectively amplify the specified *Aspergillus* species. The annealing properties of each primer on consensus sequences of *Aspergillus* species based on Tm are demonstrated in Supplementary Figure S1. Based on the plot, all primers succeed in amplifying, in silico, the consensus sequence of the corresponding species. Additionally, primers anneal to sequences of closely related species exemplified by the *A. flavus*, *A. niger*, and *A. terreus* sets under less stringent conditions (Supplementary Figure S2). These findings underscore the risk that the proposed primer sets may also detect phylogenetically close species, in addition to those they are intended to identify. However, the observed similarity and rarity of those taxa make this issue less critical for most practical applications.

## 4. Discussion

Due to their remarkable adaptability, *Aspergillus* species prevail in most environments [1]: soil, decaying vegetation, organically enriched debris, food storage rooms with high humidity, and air ventilation and water systems are some of the places where *Aspergillus* may thrive. In pharmaceutical and food industries, as well as in healthcare settings, the risk of contamination with *Aspergillus* should not be neglected. *Aspergillus* contamination of food products can lead to mycotoxicosis, which can cause a variety of adverse health effects and pose serious health threats to both humans and livestock [55,56,57]. Hospital-acquired aspergillosis is usually associated with airborne fungal contamination of the hospital environment, especially after building construction events [58].

Outside of the hospital premises, a healthy person inhales daily hundreds of *Aspergillus* conidia that are usually cleared by the immune system. However, in the case of immunocompromised patients, inhalation of conidia may cause *Aspergillus*-related diseases, including fungal infections [59]. Successful treatment of such infections is highly dependent on the early identification of the fungi involved [60,61]. The fact that certain pathogenic *Aspergillus* strains such as *A. fumigatus* and *A. terreus* have shown resistance to anti-fungal drugs such as amphotericin b and various azoles underscores the need for a straight-forward molecular tool to identify the *Aspergillus* species present in a clinical sample [23,62,63,64,65,66,67,68].

Today, a combination of methods is used to diagnose *Aspergillus*-related diseases. The imaging tests include chest X-ray or CT scan [58]. The presence of aspergillus filaments can be identified though staining of the patient’s sputum; the specimen is then placed in culture to confirm diagnosis [69,70]. Skin testing and blood tests screening for IgE levels may be helpful in confirming allergic bronchopulmonary aspergillosis [71]. In some cases, a biopsy is required; examining a lung or sinus tissue sample under a microscope may be necessary to confirm a diagnosis of invasive aspergillosis [59,72]. In recent years, real-time PCR assays have become a very popular and accurate method for surveillance or diagnosis of *Aspergillus* [73] in patients’ biological fluids, food, or environmental swabs. Most of the assays developed so far rely on hybridization probes to improve specificity. Such approaches not only have a relatively higher cost compared to the reported methodology and but also do not allow the identification of the *Aspergillus* species present in the samples [74].

In this study, we have developed a straight-forward, sensitive, rapid, and cost-effective multiplex single-color real-time PCR method combined with melting curve analysis that can detect and identify the five most pathogenic *Aspergillus* species in a single reaction. The combination of multiplex qPCR with melting curve analysis requires only a single-color reaction, thus obviating the design and use of expensive specific probes and time-consuming gel electrophoresis to detect the amplicons. Multiplex reactions simplify the analysis and further reduce the overall cost, as all five different species can be identified in a single reaction. Logotheti et al. and Erali et al. have already described a rapid multiplex PCR method for the identification of *A. fumigatus*, *A. flavus*, *A. terreus*, and *A. niger* [75,76]. However, not only can the described assay identify *A. nidulans*, but it is more straightforward as only one consensus reverse primer is included in the reactions. Moreover, in our protocol, diagnostic results are obtained in less than one and a half hours as our assay is utilizing real-time PCR and melting curve analysis.

The primers proved highly specific among the *Aspergillus* species we tested, as in all cases DNA was amplified exclusively from the targeted species. In addition, in silico analysis evaluated primers for selective *Aspergillus* species detection. Primers were successfully predicted to amplify target species, but also closely related rare ones. While not critical for most applications, sequencing analysis may be required for definitive identification. Conversely, we could regard this issue as a potential advantage of the method if we wish to detect any *Aspergillus* species of the same section as the five species studied here. In vitro, the described methodology is quite sensitive: 1 pg DNA is sufficient for the detection of *Aspergillus* species even in low-quality DNA samples. During the simulation of co-infection scenarios by combining pairs of DNA templates (Supplementary Table S1), we observed that species with higher primer melting temperature (Tm) values exhibited dominance and suppressed detection of other species, unless DNA from less potent species was present at a higher relative concentration. In conclusion, based on our analysis, we strongly recommend that when a positive result is obtained, a second multiplex should be performed omitting the primer corresponding to the species already detected in the first reaction. This strategy will allow detection and identification of co-infecting species that otherwise would possibly be masked. Hence, our method could be very useful in screening and surveillance procedures.

To our knowledge, this is the first time that a multiplex qPCR melting curve analysis method was designed for the simultaneous detection and identification of the five most common pathogenic *Aspergillus* species. This novel assay has high specificity, high sensitivity, and low cost, and it is fast and straight-forward to interpret. Thus, we suggest this method for monitoring and surveillance of putative contamination from *Aspergillus* in industry and health care.

## Figures and Tables

**Table 1:** Sequences and Tm of oligonucleotides utilized for the identification of *Aspergillus* species in a multiplex qPCR reaction.

Name	Sequence (5′-3′)	Location	Amplicon Size (bp)	Primer Tm (°C)
*A. niger* Forward	CGTTTTCCAACCATTC	ITS2	58	46.6
*A. flavus* Forward	CTTGCCGAACGCAAATCAATC	ITS2	65	57.9
*A. nidulans* Forward	TTTGTCACCCGCTCGAT	ITS2	98	52.8
*A. fumigatus* Forward	TATGCAGTCTGAGTTGATTATCGT	ITS1	371	56.9
*A. terreus* Forward	CCGAGTGCGGGTCTTTA	ITS1	554	55.2
Universal Reverse	GTTCAGCGGGTATCCCTA	LSU	-	58.2

## Data Availability

Not applicable.

## Supplementary Materials

The following supporting information can be downloaded at: https://www.mdpi.com/article/10.3390/jof9080842/s1, Supplementary Figure S1: Scatter plot of in silico PCR results for each primer set, with the two axes representing different properties useful in species identification; Supplementary Figure S2: Heat map showcasing the phylogenetic clustering of the in silico PCR results.; Supplementary Table S1: (A) Detectability of *A. fumigatus*, *A. flavus*, *A. nidulans*, and *A. niger* when co-occurring with *A. terreus*. (B) Detectability of *A. terreus*, *A. flavus*, *A. nidulans*, and *A. niger* when co-occurring with *A. fumigatus*; Supplementary Material S1: *In silico* evaluation of intra-species sensitivity of the *A. nidulans*-specific detection
