# Supplementary material for: A Multiplex PCR Melting-Curve-Analysis-Based Detection Method for the Discrimination of Five Aspergillus Species
Source: J Fungi (Basel). 2023 Aug 11;9(8):842. doi: 10.3390/jof9080842 (PMC10455196; doi:10.3390/jof9080842)
Supplement: Supplementary file 1 [file jof-09-00842-s001.zip › Supplementary Material S1.pdf]

Supplementary Table S1. (A) Detectability of *A. fumigatus, A. flavus, A. nidulans*, and *A. niger* when co-occurring with *A. terreus.* ***(B)*** Detectability of *A. terreus, A. flavus, A. nidulans*, and *A. niger* when co-occurring with *A. fumigatus.* Reactions were performed under three different ratios of the "co-infecting" species as follows: in the "1:10" column 100 pg of *A. terreus* or *A. fumigatus* was mixed to 1 ng of one of the others, in the "1:1" column equal amount of each species was mixed, and in the "10:1" column 1 ng of *A. terreus* or *A. fumigatus* was mixed with 100 pg from one of the other species. The term "traceable" denotes the ability of the assay to detect the co-infecting species while the term "untraceable" denotes the inability of the method to detect the co-infecting species.

A.	***A. terreus* (x)**	**1:10**	**1:1**	**10:1**
	A. niger	traceable	traceable	untraceable
	A. flavus	traceable	untraceable	untraceable
	A. nidulans	traceable	traceable	untraceable
	A. fumigatus	traceable	untraceable	untraceable

B.	***A. fumigatus*(x)**	**1:10**	**1:1**	**10:1**
	A. terreus	traceable	traceable	untraceable
	A. flavus	traceable	traceable	untraceable
	A. nidulans	traceable	traceable	untraceable
	A. niger	traceable	untraceable	untraceable

Supplementary Material S1

***In silico* evaluation of intra-species sensitivity of the *A. Nidulans*-specific detection primer**

In order to examine the sensitivity of the *A. nidulans* primer set, a thorough investigation was performed using publicly available sequences. Specifically, sequences including the ITS and LSU genetic loci were downloaded from the NCBI-GenBank database (release 256.0) using the following search query: *"Aspergillus nidulans"[Organism] AND ("ITS2"[All Fields] OR "ITS1"[All Fields] OR "ITS"[All fields]) AND ("LSU"[All Fields] or "28s"[All fields])*. In total, 87 sequences were retrieved, as listed below. However, multiple sequence alignment utilizing the MUSCLE v5 algorithm indicated the presence of low quality sequences. For this reason, quality control was performed using the BLAST+ software suite (v2.14.0). Each sequence was tested against the consensus sequence for *A. nidulans*, created as mentioned above. The quality of each sequence was evaluated using the calculated bit-score as a metric. Sequences with a bit-score < 700 were considered as non-representative, and consequently removed.

The ability of the primer to bind to the remaining 84 sequences was assessed using ThermonucleotideBLAST. Options were adjusted to only provide a positive hit if the primer sequence contains a maximum of 2 mismatches with the template, with none of them appearing in the final 3′ base. The results of this analysis indicate that all publicly available sequences for *A. nidulans* were sufficiently recognized by our proposed primer set.

Table of all accession ids, for the *A. nidulans* sequences retrieved from GenBank.

EF652488.1	KP131596.1	OW987672.1	MH329785.1	KC175555.1	LC482120.1	KT315496.1
EF652427.1	OW987706.1	OW986555.1	KY046245.1	OW983182.1	LC519919.1	FJ878646.1
EF652458.1	OW982578.1	AF138289.1	EF121334.1	FR733846.1	EF567973.1	JN887694.1
AJ937756.1	OW983229.1	FJ878647.1	KC175549.1	OW982577.1	EF567976.1	AF078898.1
GU205096.1	OW988284.1	KP131594.1	HG964343.1	AM158200.1	EF567977.1	AF078899.1
JN672587.1	AY452983.1	OW987586.1	NR_133684.1	AM158201.1	KC175550.1	EU982031.1
FJ878645.1	KX690140.1	KU866599.1	HQ026740.1	AM158202.1	LN808880.1	LN808965.1
FJ878643.1	KP131592.1	KU866605.1	KP131595.1	AM158228.1	LM653124.1	U93686.1
FJ878644.1	KP131593.1	KU866613.1	FR733841.1	AM158230.1	KU377335.1	U03521.1
GQ461904.1	OW983352.1	KU866627.1	KY046246.1	AM158231.1	EF567974.1	
AY373888.1	OW984112.1	KU866629.1	KU935667.1	AM158232.1	KJ775510.1	
FJ878641.1	KU687803.1	KU866630.1	KY046247.1	AM176708.1	KJ775511.1	
FJ878642.1	OW982350.1	KU866638.1	OW982707.1	FR733847.1	EF567975.1	
