# Supplementary figures and images for: A Multiplex PCR Melting-Curve-Analysis-Based Detection Method for the Discrimination of Five Aspergillus Species
Source: J Fungi (Basel). 2023 Aug 11;9(8):842. doi: 10.3390/jof9080842 (PMC10455196; doi:10.3390/jof9080842)

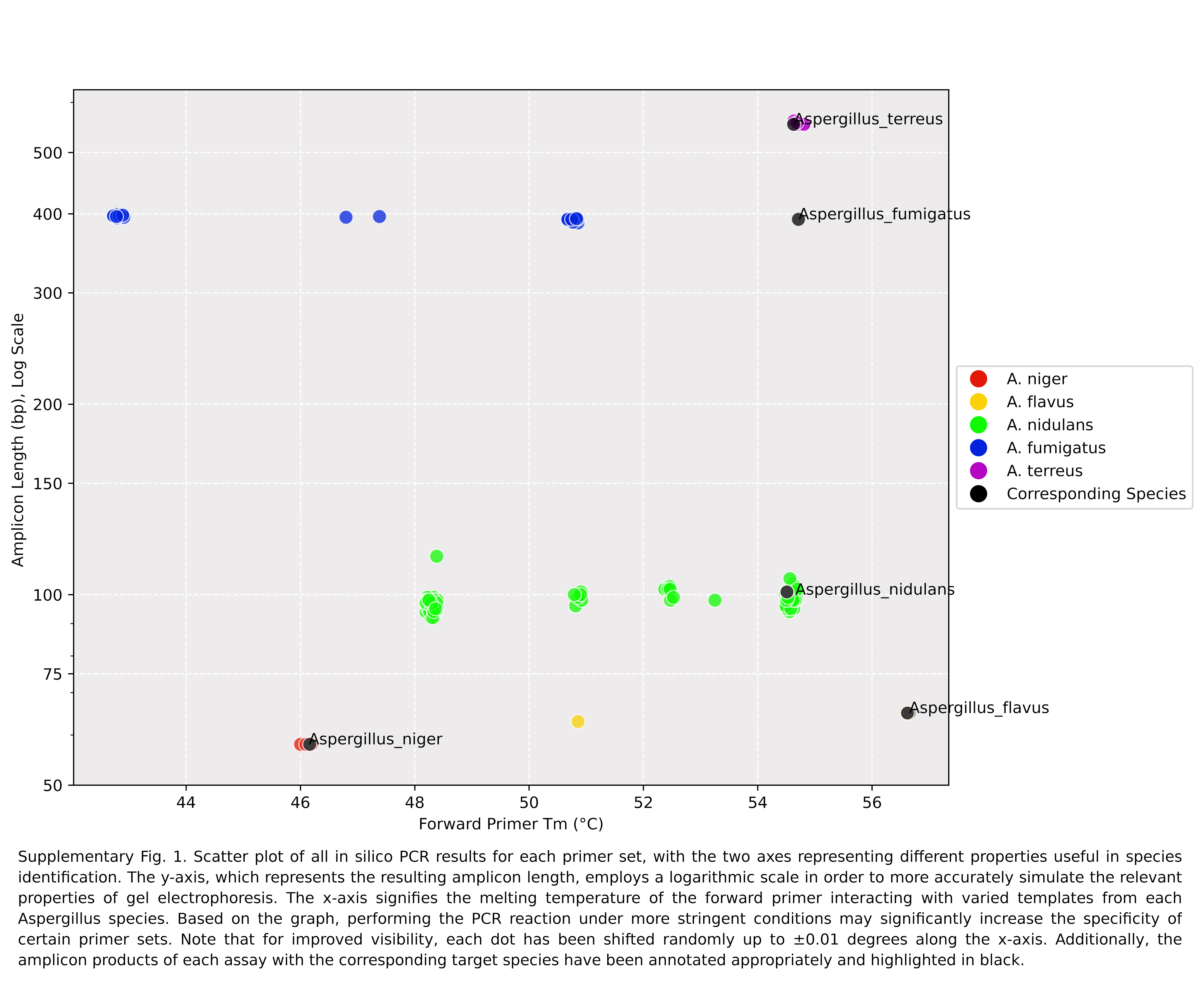

Supplement: Supplementary file 1 [file jof-09-00842-s001.zip › Supplementary Figure S1.jpg]
